# Hypodermic Injection of Ergotin in Hemorrhage

**Published:** 1872-12

**Authors:** 


### HYPODERMIC INJECTION OF ERGOTIN IN HEMORRHAGE.

In the *London Practitioner*, of December, Dr. C. Currie Ritchie, details a number of cases in which sudden arrest of hemorrhage followed the hypodermic injection of ergotin. He found Langenbeck's formula the best in practice. The solvent used in this is equal parts of glycerine and rectified spirits. The dose used was from three to five grains of the ergotin. The following is a condensed account of one of his cases: Mrs. D., aged sixty, had, during the two preceding days, been constantly spitting blood, except during an intermission of six hours. Ergot had been given by the stomach without benefit. At the time of the injection (five grains) she was expectorating blood profusely. The only physical signs were a patch of dullness, about the size of the palm, over the left back, between the angle of the scapula and the spinal column, with slightly increased vocal resonance, and accentuation of the second sound of the heart. There was obsolutely no hemorrhage after the injection.

In the *British Medical Journal*, June 3, 1871, Dr. William Allan Jamieson details a case in which repeated hemorrhage from the lungs was arrested, each time of its occurrence, by the injection of five grains of ergotin, dissolved in ten minims of water, into the cellular tissue of the arm.—*Louisville & Richmond Med. Journal.*

